# How incremental video training did not guarantee implementation due to fluctuating population prevalence

**DOI:** 10.1136/bmjoq-2018-000447

**Published:** 2019-05-04

**Authors:** Peter Vink, Bart Torensma, Cees Lucas, Markus W Hollmann, Ivo N van Schaik, Hester Vermeulen

**Affiliations:** 1 Neuro and Head/Neck, Amsterdam University Medical Center, location Academic Medical Center, Amsterdam, The Netherlands; 2 Omni Cura Nursing Teaching Research, Amsterdam, The Netherlands; 3 Department of Anesthesiology, Leids Universitair Medisch Centrum, Leiden, The Netherlands; 4 Department of Clinical Epidemiology, Biostatistics and Bioinformatics, University of Amsterdam, Amsterdam, The Netherlands; 5 Department of Anesthesiology, AMC, Amsterdam, The Netherlands; 6 Department of Neurology, AMC, Amsterdam, The Netherlands; 7 Radboud Institute for Health Sciences, Scientific Center for Quality of Healthcare (IQ Healthcare), Radboud UMC, Nijmegen, The Netherlands

**Keywords:** pain, nurses, implementation, quality improvement

## Abstract

Patients with stroke admitted at the neurology/neurosurgery ward of the Academic Medical Centre in Amsterdam, The Netherlands, may experience problems in communication, such as aphasia, severe confusion/delirium or severe language barriers. This may prevent self-reported pain assessment; therefore, pain behaviour observation scales are needed. In this project, we therefore aimed to implement the Rotterdam Elderly Pain Observation Scale (REPOS) by video training.

We used a stepped-wedge cluster design with clusters of four to five nurses with intervals of 2 weeks, for a total study duration of 34 weeks. Primary endpoint was the proportion of shifts in which nurses used the REPOS when caring for an eligible patient. A questionnaire was send biweekly to assess self-perceived competence and attitude on pain measurement in patients able or unable to self-report pain intensity. No other strategies were used to promote the use of the REPOS.

Though the proportion of shifts in which trained nurses cared for eligible patients increased from 0% at baseline to 83% at the end of the study, the proportion of cumulative shifts where the REPOS was used decreased from 14% to 6%, respectively. Process evaluation suggests that this decrease can (in part) be attributed to low and varying prevalence of eligible patients and opportunities for practice. In total, 24 (45.3%) nurses had used the REPOS at least once after 34 weeks, with a median of two times (1–33). Nurses perceived themselves 'competent' to 'very competent' in pain behaviour observation. There was no negative attitude towards pain measurement.

This study shows that education alone may not be effective when implementing a pain behaviour observation scale for non-communicative patients with Acquired Brain Injury. Individual motivation of health professionals and individual patient factors may be of influence for the use of the REPOS.

## Introduction

### Problem

Even in modern medicine, stroke has a severe impact on patients all over the world. While mortality rate is dropping and both primary prevention and acute treatment have improved, it is estimated that from 2025, Europe will count 1.5 million new patients with stroke per year due to the ageing population.^1 2^ Among the many different effects and complications of stroke, there are some that prevent adequate communication, such as severe aphasia, confusion and delirium.

Aphasia is present in about one-third of all patients with acute stroke in varying forms and severity.^3–5^ Though evidence on the prevalence of severe confusion is not available, the prevalence of a formally diagnosed delirium lies around 10%.^6 7^ With about 41 300 patients with stroke per year in the Netherlands, this would annually mean 13 800 patients with aphasia and 4130 patients with delirium during the acute phase of stroke.^8^

Besides these effects originated by the stroke itself, the Academic Medical Centre (AMC) in Amsterdam, The Netherlands, often comes across patients unable to speak Dutch. Amsterdam hosts many multi-lingual people of different backgrounds. There are about 180 different ethnicities in the city and at least 35% of the population has a non-Western migration background.^9^ It is therefore not uncommon that patients are admitted with severe language barriers for nurses who are mainly Dutch native speaking.

At the neurology/neurosurgery nursing ward of the AMC, the ‘AMC Neurocentre’, patients are often admitted with one or more of the above-mentioned communication disorders. This may interfere with common nursing practices or communication, in particular the measurement of pain intensity. Addressing pain is considered as being fundamental or basic nursing care and essential for delivering high care quality.^10^ The nurses of the ‘AMC Neurocentre’ therefore asked for (video) training to provide the skills and knowledge that is needed for pain behaviour observation. The team consists of approximately 55 nurses with different levels of education (Associate and Bachelor degree) and experience in neuroscience nursing.

### Available knowledge

According to international guidelines, self-reported pain instruments are considered the best possible method. Commonly used instruments are the Numerical Rating Scale (NRS), Visual Analogue Scale, Wong-Baker Faces Pain Scale-Revised and the Verbal Rating Scale.^11 12^ In some cases of patients with stroke with one or more of above-mentioned communication problems, none of these instruments work sufficiently well and nurses must rely on pain behaviour observation scales to adequately assess (potential) pain.

The Rotterdam Elderly Pain Observation Scale (REPOS) has been developed in 2009 for older patients incapable of reporting pain themselves. It is a Dutch scale with 10 behaviours that may indicate the presence of pain.^13^ As patients with stroke and specifically patients with aphasia or delirium tend to be older, this scale seems appropriate for the use in acute stroke care.^7 14 15^

### Rationale

Though single-component (educational) implementation strategies are generally considered less effective, there is also no compelling evidence that multifaceted strategies are more effective in changing healthcare professionals’ behaviour.^16–18^ Training with patient videos (focused both on knowledge and skills) has proven to be effective for implementation of pain behaviour observation scales.^19^

Training the entire ‘AMC Neurocentre’ nursing team at once is costly, logistically complicated and may not provide a sustainable solution that withstands regular changes within the team. We therefore chose a more gradual educational strategy by using a stepped-wedge cluster design with parts of the nursing team as clusters. We hypothesise that the risk of contamination from this design will cause the number of nurses using the REPOS to increase faster than the number of nurses receiving training and may be used as a potential method of implementation itself.^20^

### Aims

In this evidence-based quality improvement study, we aim to evaluate (a) whether an educational strategy can increase the use of the REPOS in patients with severe aphasia, confusion or language barriers and (b) whether the risk of contamination from a stepped-wedge cluster design within a nursing team has an effect on the speed of implementation.

We consider the implementation successful whether at the end of the study…

- ….the REPOS is used in ≥85% of the shifts in which nurses care for eligible patients
- …pain assessment is compliant (≥1 REPOS measurement per 12 hours) in ≥85% of patient days.

## Methods

### Context

The ‘AMC Neurocentre’ is part of a tertiary care setting with regional responsibilities. It provides specialised care in cerebrovascular diseases such as intra-arterial thrombectomy and coiling of intracranial aneurysms. It operates 20 regular nursing beds and nine beds on the Brain Care Unit, a Stroke Unit for acute cerebrovascular care. A multidisciplinary team is available, consisting of neurologists, neurosurgeons, nurses, nursing aids, physiotherapists, occupational therapists and speech therapists. In 2017 the unit had 129 admissions per month on average, of which 108 (83%) had a length of stay longer than 24 hours. The average length of stay was 7.2 days.

#### Participants

The participants of this study were registered nurses working at the ‘AMC Neurocentre’, with either an Associate (or similar) or Bachelor degree in nursing. Nurses who were not involved in nursing care activities, for example due to illness, or who had a temporary contract (<two months) were not included in the study.

### Target(s)

#### Recommendation

The instrument to be implemented is the REPOS, a pain behaviour observation tool developed for elderly patients unable to use self-reported pain instruments.^13^ As of February 2017, pain behaviour observation is part of the local hospital protocol and the REPOS is indicated for patients admitted to general nursing wards who are unable to self-report pain intensity, though still able to make verbal or non-verbal contact with health professionals. A flowchart is part of the local hospital protocol to help nurses decide on the use of pain behaviour observation tools.

The REPOS consists of 10 behavioural items that are associated with the presence of pain, each worth one point. The nurse observes the patient for a minimum of 2 min, at least twice a day, once during ambulation or nursing care activities and once during rest. If the REPOS shows a score of 2 or higher, the nurse will assess possible causes for the observed behaviour and provide a second pain score that reflects their clinical judgement. This is a NRS score where zero indicates that the nurse assumes there is no pain present that causes the behaviour and 10 indicates that the nurse assumes the worst imaginable pain is present. A list of other possible causes of the observed behaviour (hunger, full bladder, fear) aids the nurse in choosing an appropriate NRS. The REPOS is documented in the electronic patient file and evaluated daily during rounds with the physicians.

The aim of pain behaviour observation with the REPOS is to provide insight in pain intensity for non-communicative patients and eventually more adequate pain treatment. As the treatment of pain is dependent on adequate pain assessment, this study focuses on the implementation of the REPOS and not the treatment of pain itself.

### Intervention

#### Educational strategy

The initiative for video training was based on desires of the nursing team to be classically trained and approved by the staff advisor on education prior to the start of the study. Every 2 weeks clusters of four to five nurses received a standardised training for 45 min at two consecutive days, with a total of 14 clusters. Nurses were allocated to a cluster based on the planned working schedule for each time period. Clusters were therefore random at first, but as the study progressed the untrained nurses would deliberately be scheduled to work on days of the video training.

The video training started with a short introductory presentation, explaining the aim of pain behaviour observation, the content of the local hospital protocol and how to use and document the REPOS. An online training module with 12 practice videos of real patients, developed by the designers of the REPOS, was then used to familiarise the nurses with the scale.^21^ During training, each of the four to five nurses observed each video and assessed pain behaviour with the REPOS individually, after which an inter-rater agreement (Fleiss’ kappa, fixed marginal) was calculated for the cluster.^22^ If there was less than substantial agreement (κ≤0.60), observation differences were discussed and the video was repeated until agreement was substantial or higher. Videos were always alternated with a video of a different patient before repeating, to avoid repetition learning. Training was provided by the same trainer (PV), who used a checklist to ensure each cluster received the same tips on the use and documentation of the REPOS. For more information about the training and responses, see the process evaluation in the Results section.

#### Motivational strategy

No motivational strategy was used to promote the use of the REPOS. Every 2 weeks however, alternating the training periods, a questionnaire was send as part of the study. This may be considered as a repeated reminder.

### Measures

Data were obtained from patient data files by the first author as part of a regular quality evaluation (PV). Besides the indication for pain behaviour observation, no patient characteristics were collected.

#### Primary outcomes

The primary outcomes reflect behaviour of nurses, as this best reflects actual implementation.

- Proportion of shifts in which nurses used the REPOS when caring for an eligible patient.
- Proportion of patient days at which pain assessment was compliant to local hospital protocol (≥1 REPOS measurement per 12 hours)

#### Secondary outcomes

Secondary outcomes were as follows: (a) the self-perceived competence in pain behaviour observation of patients able and unable to self-report pain and (b) the attitude towards pain measurement. The self-perceived competence was measured with a 4-point Likert scale, where a higher score meant the nurse felt more competent (very incompetent, incompetent, competent and very competent).

- Recognising pain in patients that can communicate well.
- Measuring pain intensity in patients that can communicate well.
- Recognising pain in patients that cannot communicate well or are (severely) confused.
- Measuring pain intensity in patients that cannot communicate well or are (severely) confused.

Attitude was measured with an adaption of the Negative Pain Belief Scale (NPBS) by Shugarman *et* *al* (2010), consisting of four questions with a 5-point Likert scale.^23^ A higher cumulative score meant a more negative attitude towards pain measurement. See online supplementary appendix 1 for more information.

10.1136/bmjoq-2018-000447.supp1 Supplementary data

Data for secondary outcomes were collected with a questionnaire that was send every 2 weeks by email. To promote completion of the survey a reward, in form of a gift card worth 5–25 euro, was allotted among every 10 completed questionnaires and reminders were send out daily for 1 week.

#### Process evaluation

To assess mechanism(s) through which the implementation strategy (gradual video training) may or may not work, the following data were collected for process evaluation:

- Percentage of trained nurses (absolute numbers and full-time equivalent).
- Proportion of shifts during which trained nurses cared for eligible patients.
- Number of trainings completed, including video's shown and repeated.
- Experienced workload as a barrier in pain assessment.
- Nursing views on their influence on pain treatment.
- Process data are used for a side-by-side analysis of the primary outcome.

Verbal responses of the participants were gathered by the trainer (PV) both during and after training to provide qualitative insights to above-mentioned data. At the end of the second day of training, nurses were asked an open-ended question how they experienced the video training.

### Statistical analysis

For analyses, we used descriptive statistics and inferential statistics. All data were first tested for normality by a Kolmogorov-Smirnov test, a Q–Q plot and Levene’s test.

Categorical variables were expressed as n (%). Continuous normally distributed variables were expressed by their mean and SD, not normally distributed data by their median and minimum and maximum range for skewed distributions. To test groups, categorical variables were tested using the Pearson’s χ^2^ test or Fisher’s exact test, when appropriate. Normally distributed continuous data were tested with the independent samples Students’ t-test and in case of skewed data, with the independent samples Mann-Whitney U test. Data were analysed with R Statistics (V.1.0.153) and SPSS (V.24.0, SPSS, Chicago, IL, USA).

### Sample size calculation

Methods of Hussey and Hughes (2007) were used for power calculation for the main outcomes, with 16 time periods and 13 clusters with at least one observation per time period. With an alpha of 0.05, a power of ≥0.8 can be obtained for increase in proportions from 0% to ≥15% or 15% to ≥42%.^24^

### Ethical considerations

The research protocol was approved by the medical ethical committee of the AMC Hospital on January 6, 2017. Data were collected for routine quality control by PV in the role of staff advisor on quality and patient safety. Other than the indication for pain behaviour observation and the duration of communication disorders, no individual patient data were collected.

## Results

The study was conducted in 2017 from February 27th to September 29th. A total of 835 individual patient files were evaluated for patients eligible for pain measurement with the REPOS. This resulted in the inclusion of 88 patient files (639 patient days) for analysis of our primary endpoint. The frequencies of indications for the use of the REPOS are shown in table 1. For reporting, the Standards for Quality Improvement Reporting Excellence 2.0 was used.^25^

**Table 1:** Frequencies of indications for the use of the REPOS

Patients unable to self-report pain intensity total)	88 (100%)
* * *D* *ue to aphasia*	41 (46.6%)
* * *D* *ue to confusion/delirium*	15 (17.0%)
Due to language barrier	14 (15.9%)
Due to a combination	18 (20.5%)
Aphasia and confusion	*6* (*6.8%*)
Aphasia and language barrier	*6* (*6.8%*)
Confusion and language barrier	*5* (*5.7%*)
Aphasia, confusion and language barrier	*1* (*1.1%*)
**Duration of communication disorder (days**)	**3.5 (1.0–** **35.0**)
**REPOS measurements required (per patient**)	**4 (1–** **35**)

REPOS, Rotterdam Elderly Pain Observation Scale.

During the study period, 68 nurses were employed at the Neurocentre, of which 62 were included in the study. Most nurses (51.4%) had an Associate degree (or similar) and 52.9% had less than 5 years of experience in neuroscience nursing. Further characteristics are shown in table 2. A total of 50 nurses received the training, resulting in 90% of the employed nurses being trained at the end of the study.

**Table 2:** Nurse characteristics

Nurses employed (per day, median, min-max)	55 (52–58)
Working contract (median hours per week, min-max)	32 (20–36)
Full-time equivalent (median, min-max)	32.0 (31.4–32.2)
Number of shifts worked in care (median, min-max)	79 (4–129)
Years of experience in neuroscience nursing	
<1 year	16 (23.5%)
1–5 years	20 (29.4%)
5–10 years	10 (14.7%)
>10 years	22 (32.4%)
Level of education	
Associate degree (or similar)	35 (51.4%)
Bachelor degree	32 (45.6%)
Master degree	1 (1,4%)

### Training

The proportion of shifts in which trained nurses cared for eligible patients increased significantly from 0% at start of the study to 83% at the end of the study (p value<0.001).

#### Process evaluation

All trainings were held at the end of the day shift and were held on two consecutive days, except for cluster 10 in which training days were 1 day apart. Nurses were interested in the training and eager to learn about the REPOS, often asking the trainer when they would be scheduled for training. Some clearly stated they were waiting for the training before they would use the REPOS. Reactions to the training were generally positive. Frequently mentioned quotes during the debriefing of the video training were as follows:

- ‘The REPOS is not complicated, but it requires some practice’
- ‘I am able to use the REPOS right away’
- ‘The videos are good for practice, because we can rewind and pause the behaviour’
- ‘It is difficult to rely on something (ie, pain behaviour observation) that is so subjective’
- ‘I find it hard to determine when I should use the REPOS’

Participants found the nuances in word choice (ie, pain behaviour) the most difficult and much of the discussions during training were on the interpretation of the pain behaviours as described by the original designers of the scale. This was an important aspect of the training though, as it contributed to good inter-rater agreement.

Planning of biweekly training based on existing working schedules proved logistically complex. As more nurses were trained, it became more difficult to find 2 days were enough untrained nurses would be present. It also proved difficult to start the training on time, as it was planned at the end of the day shift. The training had a median duration of 85 min (85–90) per training, during which a median of 14 videos (10–15) were observed. Videos were played one to three times (median 1) before a substantial inter-*rater agreement (kappa ≥0.67) was achieved.

### Nurse behaviour

The REPOS was documented 138 times for 36 (41%) eligible patients, of which 48 (35%) were measured by trained nurses. The proportion of nurses that at some point during the study period used the REPOS at least once for eligible patients increased gradually from 4 (7,4%) to 24 (45.3%) at the end of the study (p value<0.001), with a median of two times (1–33). Four nurses were responsible for 62% of all REPOS measurements and 51% of all REPOS measurements were performed in 8% (n=7) of all eligible patients.

In total, there were 96 nursing shifts (5,8%) with at least one REPOS measurement. Overall, the proportion of cumulative shifts in which the REPOS was used (when required) decreased from 14% at baseline to 6% at the end of the study (p value<0.01). The OR of cumulative shifts with versus without a REPOS measurement compared with baseline increased during time period 1 through 6, but declined again after that (table 3). These changes in OR were statistically significant from baseline for each time period, with the exception of T6 (10–12 weeks).

**Table 3:** Proportion of shifts with and without REPOS measurements and OR when compared with baseline

Time period	Cumulative shifts with REPOS	Cumulative shifts without REPOS	OR (95% CI) when compared with baseline	P value
**1**	13	80	–	–
**2**	14	243	0.36 (0.15 to 0.86)	0.012*
**3**	28	419	0.41 (0.19 to 0.90)	0.017*
**4**	36	547	0.41 (0.20 to 0.87)	0.015*
**5**	44	651	0.41 (0.21 to 0.88)	0.016*
**6**	67	814	0.51 (0.26 to 1.04)	0.044*
**7**	78	984	0.49 (0.25 to 1.00)	0.041*
**8**	81	1056	0.47 (0.24 to 0.97)	0.024*
**9**	83	1087	0.47 (0.24 to 0.96)	0.024*
**10**	86	1199	0.44 (0.23 to 0.90)	0.019*
**11**	90	1340	0.41 (0.21 to 0.84)	0.009**
**12**	93	1433	0.40 (0.21 to 0.81)	0.007**
**13**	94	1471	0.39 (0.20 to 0.80)	0.007**
**14**	94	1479	0.39 (0.21 to 0.80)	0.007**
**15**	95	1516	0.39 (0.20 to 0.78)	0.006**
**16**	96	1569	0.38 (0.20 to 0.76)	0.006**

*P value<0.05, **p value<0.01.

REPOS, Rotterdam Elderly Pain Observation Scale.

In 16 (2,5%) patient days, the REPOS measurement was compliant to local hospital protocol (≥1 REPOS measurement per 12 hours). The proportion of compliant patient days did not change significantly over the course of the study, ranging from 0% to 16% (in T6).

#### Process evaluation

**Figure 1:**
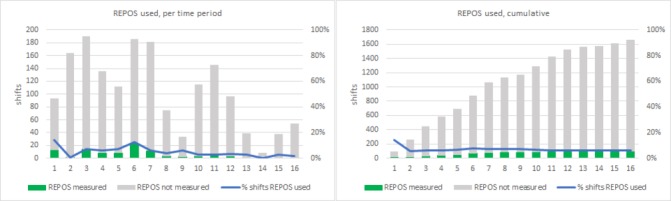
Green (part of) column: REPOS measured. REPOS, Rotterdam Elderly Pain Observation Scale.

During the study, all nurses cared at least one shift for eligible patients, with a median of 27 shifts (1–57). The chance of exposure to an eligible patient fluctuated due to varying prevalence and started to decrease drastically after T7. In the first three time periods, an average of 166 REPOS measurements was required, whereas in the last three time periods an average of 33 REPOS measurements were required. In time period 14 (18–22 weeks), only eight REPOS measurements were required. This decrease in chance for practical application of acquired skills and knowledge may (in part) have attributed to decline of the use of the REPOS. The number of required and performed REPOS measurements, both cumulative and per time period, are shown in figure 1.

### Secondary outcomes

On average, the questionnaire was completed by 55% of the employed nurses per time period of 2 weeks. The completion rate decreased over the duration of the study from 76% to 39% (p value<0.001).

#### Self-perceived competence

At the start of the study, 97% of the nurses that completed the questionnaire considered themselves 'competent' to 'very competent' in recognising and measuring pain in patients who are able to communicate adequately. This increased to 100% at the end of our study. For patients that require pain behaviour observation, 86% of the nurses considered themselves 'competent' to 'very competent' at the start of the study, which increased to 93%.

#### Attitude

During the study, there was one measurement of one nurse (0.3%) with a negative attitude towards pain measurement (NPBS >12). All other time periods there was no negative attitude among nurses. The NPBS did not change significantly during the study.

#### Other

From the 361 responses to the questionnaire, the participants agreed or strongly agreed 300 (83.1%) times with the statement ‘my colleagues find pain assessment an important part of the nursing profession’. In 94 responses (17.7%), the participants agreed or strongly agreed with the statement ‘I am unable to measure pain in patients who can’t communicate well or are severely confused due to lack of time or increased workload’. In 87 (24.1%) of the responses, they were neutral on this statement and in 180 (49.9%) they disagreed or strongly disagreed. For the statement ‘nurses have no influence on the treatment of pain’, nurses disagreed in 349 (96.7%) of their responses.

## Discussion

In this evidence-based quality improvement study, we aimed to evaluate an educational implementation strategy to implement a pain behaviour observation scale. For this purpose, we used a stepped-wedge cluster design within one nursing team, expecting the risk of contamination to be of positive influence on the speed of implementation. Despite a consistently executed video training, where inter-rater agreement was obtained within clusters, our aims for implementation were not achieved. Though there was a significant increase in the proportion of trained nurses that cared for patients that required pain behaviour observation with the REPOS, the actual use, which is equivalent to behaviour in our view, decreased during the study period.

We chose this single-component implementation strategy because our nurses explicitly asked for education, which is quite common among healthcare professionals when there is a need to change practice or implement scientific innovations. Though we are aware of the positive impact of motivational strategies, we deliberately wanted to evaluate the effect of education alone and to determine whether we should acknowledge healthcare professionals’ wishes for educational strategies in future implementation projects.

The process data in this study provides insight in mechanisms that may have prevented nurses from using the REPOS more frequently. The results of the NPBS show, for example, that there was no negative attitude towards pain measurement in general and pain behaviour observation in particular. The questionnaire results show that lack of time or increased workload was not a barrier for pain measurement or behavioural pain observation. This is in accordance with studies on ‘care left undone’ during nursing shifts, which show that pain management and treatment are least likely to be reported as missed.^26 27^

Data on the provided training show that limited repetition of videos (maximum three times) were needed to acquire a substantial agreement among the nurses. This indicates that the videos were suited for acquiring basic skills to use the REPOS for pain behaviour observation. In order to maintain and improve these skills, it seems that practicing with actual patients is important. In at least six time periods, both the number of individual patients and the number of required REPOS measurements were very low, down to 4 required REPOS measurements per week in time period 14. During the study, the chance for nurses to be exposed to eligible patients decreased and as such the chance of nurses practicing their acquired skills decreased as well. After video training, the nurses were eager to practice, but for some nurses several weeks passed before they got the first opportunity to use the REPOS.

It is noticeable that most REPOS measurements were performed by the same group of nurses. These nurses used the REPOS before training and continued to do so after training. This may indicate that individual motivation is more important than mere training.

The qualitative insights gathered during and after training also indicate that nurses find it hard to indicate when a patient requires a REPOS measurement instead of other instruments. This is also reflected by the fact that more than half of the REPOS measurements were performed in only 8% of the eligible patients. This suggests that once a nurse starts using the REPOS others may follow, but if nobody starts using it the patient receives no form of pain assessment.

### Limitations of the study

The choice for a stepped-wedge cluster design within a single nursing team, whereby multiple measurements were done by the same nurses, has shown some limitations in this study. Due to the decrease in the actual use of the REPOS, formal analysis using a generalised mixed model or generalised estimation equation was not possible. Therefore, we analysed between and within baseline and every time point towards an allowable statistical procedure. Another limitation of this study was that actual knowledge and skills, obtained at the end of training, was not consistently measured. We are therefore unable to prove that the training itself guaranteed the skills and knowledge needed in daily nursing care. In future studies, a standardised test reflecting learning points of the training should be incorporated in the measurements. The feedback at the end of the training was generally positive, but this may have been due to interviewer bias as the evaluation was done by the trainer himself. In future, similar quality improvement studies we suggest to perform a barrier and facilitator analysis to determine both required training forms and skills measurement.

## Conclusion

This study shows that education alone may not be effective when implementing an evidence-based quality improvement. Pain behaviour observation for non-communicative patients with acquired brain injury may be more complicated than merely providing knowledge and (simulated) practice. Future implementation projects or research should include an extensive assessment of potential barriers (prevalence, chance of exposure) and facilitators in order to adequately select a motivational strategy alongside education.
