# Supplementary material for: How incremental video training did not guarantee implementation due to fluctuating population prevalence
Source: BMJ Open Qual. 2019 May 4;8(2):e000447. doi: 10.1136/bmjoq-2018-000447 (PMC6542455; doi:10.1136/bmjoq-2018-000447)
Supplement: Supplementary data [file bmjoq-2018-000447supp001.docx]

# Appendix 1

The adaption of the Negative Pain Belief Score (NPBS, including typographical emphasis (bold and italic), as used in the questionnaire.

Patients **don’t** report their pain accurately.

1. Strongly disagree
2. Disagree
3. Neutral
4. Agree
5. Strongly agree

Pain **can’t** be measured by observing a patients behaviour.

1. Strongly disagree
2. Disagree
3. Neutral
4. Agree
5. Strongly agree

Patients who **don’t** report pain *themselves* have **no** pain.

1. Strongly disagree
2. Disagree
3. Neutral
4. Agree
5. Strongly agree

Patients who **can’t** report pain themselves have **no** pain.

1. Strongly disagree
2. Disagree
3. Neutral
4. Agree
5. Strongly agree
